# Complementary multi-modality molecular self-supervised learning via non-overlapping masking for property prediction

**DOI:** 10.1093/bib/bbae256

**Published:** 2024-05-27

**Authors:** Ao Shen, Mingzhi Yuan, Yingfan Ma, Jie Du, Manning Wang

**Affiliations:** Digital Medical Research Center, School of Basic Medical Sciences, Fudan University, 131 Dong’an Road, 200032, Shanghai, China; Shanghai Key Laboratory of Medical Image Computing and Computer Assisted Intervention, Fudan University, 131 Dong’an Road, 200032, Shanghai, China; Digital Medical Research Center, School of Basic Medical Sciences, Fudan University, 131 Dong’an Road, 200032, Shanghai, China; Shanghai Key Laboratory of Medical Image Computing and Computer Assisted Intervention, Fudan University, 131 Dong’an Road, 200032, Shanghai, China; Digital Medical Research Center, School of Basic Medical Sciences, Fudan University, 131 Dong’an Road, 200032, Shanghai, China; Shanghai Key Laboratory of Medical Image Computing and Computer Assisted Intervention, Fudan University, 131 Dong’an Road, 200032, Shanghai, China; Digital Medical Research Center, School of Basic Medical Sciences, Fudan University, 131 Dong’an Road, 200032, Shanghai, China; Shanghai Key Laboratory of Medical Image Computing and Computer Assisted Intervention, Fudan University, 131 Dong’an Road, 200032, Shanghai, China; Digital Medical Research Center, School of Basic Medical Sciences, Fudan University, 131 Dong’an Road, 200032, Shanghai, China; Shanghai Key Laboratory of Medical Image Computing and Computer Assisted Intervention, Fudan University, 131 Dong’an Road, 200032, Shanghai, China

**Keywords:** multi-modality self-supervised learning, molecular property prediction, molecular representations

## Abstract

Self-supervised learning plays an important role in molecular representation learning because labeled molecular data are usually limited in many tasks, such as chemical property prediction and virtual screening. However, most existing molecular pre-training methods focus on one modality of molecular data, and the complementary information of two important modalities, SMILES and graph, is not fully explored. In this study, we propose an effective multi-modality self-supervised learning framework for molecular SMILES and graph. Specifically, SMILES data and graph data are first tokenized so that they can be processed by a unified Transformer-based backbone network, which is trained by a masked reconstruction strategy. In addition, we introduce a specialized non-overlapping masking strategy to encourage fine-grained interaction between these two modalities. Experimental results show that our framework achieves state-of-the-art performance in a series of molecular property prediction tasks, and a detailed ablation study demonstrates efficacy of the multi-modality framework and the masking strategy.

## Introduction

Accurate prediction of molecular properties is the basis for compound screening [1] and accelerates the drug discovery process [2]. Efficient molecular representation is a priority for predicting molecular properties [3]. Recently, molecular representation learning based on deep learning has been booming [4, 5]. However, since most molecular label data need to be obtained through labor-intensive and costly wet experiments [6], there is a lack of sufficient labeled molecular data, which hinders the development of deep learning methods and can lead to issues like overfitting and poor generalization [7, 8]. Self-supervised learning holds substantial research value in addressing these challenges, which involves pre-training on unlabeled data and fine-tuning with labeled data on downstream tasks. It has shown significant promise in enhancing the performance of molecular representation learning on property prediction tasks [9].

**Figure 1:**
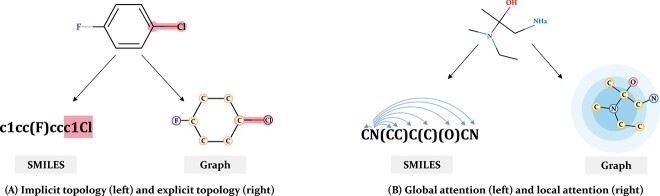
Comparison of two molecular representation modalities, SMILES and graph. (A) Illustration of the topological differences between the two modalities. SMILES represents topology implicitly, while graph displays explicit topology. (B) Difference in receptive field of networks for the two modalities. Global attention is usually used for SMILES, while local attention can be easily implemented for graph.

Molecules can be described using various modalities, such as fingerprints, sequences, graphs and more [10–12]. Our work mainly focuses on two widely used modalities: Simplified Molecular-Input Line-Entry system (SMILES) [13] and molecular graph. As depicted in Fig. 1, the same molecule can be represented using both a SMILES sequence and a graph, with each modality having its unique advantages and disadvantages. SMILES is a compact implicit representation of the molecule that excludes single-bond representation, making it well suited for rapid compound retrieval and identification [14]. Additionally, the SMILES sequence, being a text string, can be processed with Transformer-based networks well developed in the natural language processing (NLP) field for feature extraction, in which the self-attention mechanism weighs and combines information from any position in the input sequence, thereby facilitating the capture of global contextual information [11, 12]. However, SMILES representations only capture the relationships between atoms and bonds. They often struggle to capture the complex structural and topological information of molecules, such as the number and positions of rings, the length of side chains and other intricate details that can be crucial in drug efficacy prediction [15, 16]. Graph representations offer explicit portrayals of atoms, bonds and their interconnections, showcasing the topological structures of molecules [17]. They provide detailed chemical information about molecules, including attributes for each atom such as element type, charge state and stereochemistry, and attributes for each bond, such as bond type and bond length [18]. However, graph neural networks, commonly used to extract features from graphs, primarily rely on message-passing layers to gather information from neighboring nodes, emphasizing the capture of local contextual information. This can lead to a disadvantage in capturing global context information due to information decay when delivering messages between non-adjacent nodes [19]. As a result, for the same molecule, SMILES and graph encode molecular features from different perspectives, offering complementary information. The rational combination of these two modalities holds promise for enhancing molecular representation performance.

There are several existing works on multi-modality molecular pre-training [20–23]. For example, GraphMVP [20] focuses on joint pre-training with 2D graphs and 3D graphs. However, these two modalities exhibit high similarity. Additionally, this study only proved that 3D geometry complements 2D topology in downstream tasks, without proving that 2D topology complements 3D geometry. MoleculeSTM [23] focuses on molecular graphs and text descriptions, using a contrastive learning strategy to learn the consistency between the chemical structure of molecules and their textual descriptions. MOCO [24] and DVMP [21] extract features from SMILES and graph of the same molecule through two specialized encoders and then utilize contrastive learning to minimize the feature distance between different modalities of the same molecule. All these constrastive learning-based methods lack fine-grained cross-modality interactions, resulting in suboptimal performance. Therefore, to achieve better performance, the challenge of more efficiently combining these two modalities with significant differences lies in how to promote information exchange in fine-grain such as at the atom level rather than only achieving contrastive learning at the entire molecule level.

To achieve information interactions in fine-grain, it is necessary to enable the network to understand the fine-grained data in different modalities simultaneously. Recently, a series of Transformer-based methods [25–28] provide a new way to understand multi-modality data. Specifically, they utilize special encoders to map multi-modality data into a universal embedding space, where images/videos can be viewed as foreign languages for language models so that language models understand them. This inspires us to encode multi-modality data into a unified pattern so that a unified Transformer-based network can learn interaction features of different modalities. Specifically, we treat words in SMILES sequences and graph nodes as tokens [29, 30], and put them into a unified network to perform fine-grained feature interaction between the two modalities. Furthermore, to better learn complementary information, we need to develop strategies to enhance the interaction between the two modalities in pre-training. Inspired by the ‘cloze-type’ generative pre-training task in NLP, which restores the masked part of the information by learning the relationship between the features of the unmasked information, designing an appropriate masking strategy can promote the pre-training network to learn richer features. Intuitively, the information used for reconstructing the masked tokens can come from the context within the same modality, as well as information from the tokens of corresponding structures in the other modality. Therefore, we establish fine-grained correspondences between the SMILES and the graph of a molecule and mask non-overlapping parts in these two modalities to encourage the model to reconstruct the masked part of one modality with the direct information of the corresponding part of the other modality, which strengthens the interactions between the two modalities.

In this paper, we propose MoleSG, a simple yet effective pre-training framework for effectively exploring the complementary information between SMILES and graph in molecular pre-training. Our framework consists of two independent encoders to separately convert masked SMILES and masked graph of an input molecule into token embeddings. Then, we introduce a Transformer-based unified backbone network for jointly processing embeddings from both modalities to facilitate interactions between them. The embeddings from the two modalities are concatenated and inputted into the universal Transformer for joint processing and the output is used to reconstruct the original SMILES and graph by two specific decoders. Our framework is trained by reconstruction losses. Furthermore, we introduce a dedicated non-overlapping masking strategy, in which we establish the atom index correspondence between the SMILES sequence and the graph of a molecule to ensure that regions masked in SMILES and graph do not overlap. To evaluate the effectiveness of MoleSG, we conduct experiments on 14 downstream tasks related to molecular property prediction and MoleSG achieves state-of-the-art (SOTA) performance in all tasks. We also compare it with the same network pre-trained by a single modality, and the experimental results show that multi-modality training learns richer molecular representation knowledge.

Our contributions are as follows: (1) we propose MoleSG, a novel molecular pre-training framework that utilizes the complementary information of SMILES and graph representations, resulting in improved performance; (2) we introduce an innovative non-overlapping masking strategy and a unified network for handling two distinct modalities, allowing for fine-grained interaction between SMILES and graph representations and achieving better representation learning; (3) MoleSG achieves SOTA performance in a series of molecular property prediction tasks, and detailed ablation study demonstrates efficacy of the multi-modality structure and the masking strategy.

## Related work

**Molecular representation learning:** In recent years, with the development of deep learning, some learning-based molecular representation learning methods have been proposed and achieved great progress, but these learning-based methods are often limited by the availability of labeled data. To solve this problem, a series of pre-training methods [31, 32] have been proposed that can utilize unlabeled data. However, most methods only use single modality data representation such as SMILES [11, 12, 33, 34], but neglect the complementary information between the different modalities, thereby achieving suboptimal performance. As analyzed in Fig. 1, multi-modality pre-training contains more information and tends to achieve better performance, thereby having greater potential.

**Molecular multi-modality self-supervised learning:** As shown in Fig. 1, the rational combination of SMILES and graph holds promise for enhancing molecular representation performance. Most existing approaches often rely on the contrastive method, such as SMICLR [35], DVMP [21] and MOCO [24], which focus on the same two modalities as we do but they neglect the fine-grained interactions across different modalities. A concurrent work, UniMAP [36], is a generative pre-training based on mask reconstruction, but it only performs simple mask reconstruction without a specific design of masking strategy, so it still cannot fully leverage the complementary information interactions. We introduce a non-overlapping masking strategy to force cross-modality information interaction, thereby having a greater advantage.

## Materials and methods

### Framework of MoleSG

**Figure 2:**
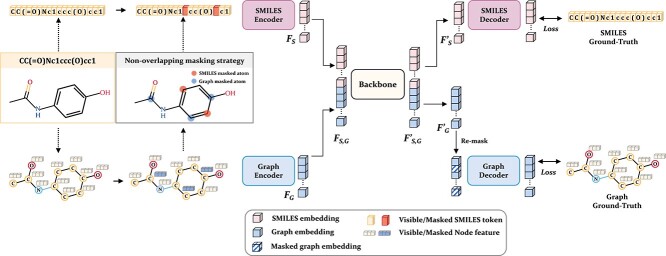
Overview of MoleSG. The SMILES sequence and the graph of a molecule are first randomly masked using the non-overlapping masking strategy. Then they are individually encoded by independent encoders, and the SMILES embeddings and the graph embeddings are concatenated and inputted into a Transformer-based backbone for joint processing. Finally, processed features belonging to each modality are decoded into token IDs and graph nodes for the reconstruction proxy task.

As shown in Fig. 2, MoleSG learns features jointly from SMILES and graph by performing masked reconstruction on both modalities with a unified feature extraction backbone network. Concretely, for a given molecule, we first convert its SMILES sequence into tokens $T_{S}$ and calculate features $V_{G}$ and $E_{G}$ for nodes and edges in the graph. During pre-training, we randomly mask some node features ${V_{G}}^{M}$ in the graph and then mask a portion of SMILES tokens ${T_{S}}^{M}$ corresponding to the remaining unmasked atoms in the graph, so that we can perform non-overlapping masking to facilitate the interaction of information between the two modalities.

During pre-training, we employ a symmetric joint encoder–decoder framework to perform further feature extraction. The framework consists of two independent branches for the two modalities and a shared backbone for feature fusion. The independent encoder branches encode the data of two different modalities into a unified form i.e. embedding, which is suitable for understanding by a Transformer-based backbone [29, 30]. The shared Transformer-based backbone can learn the dependencies between atoms within and across the modalities and output features for the subsequent independent decoders. Finally, the SMILES Decoder and the Graph Decoder reconstruct the original SMILES sequence and graph based on the output of the backbone. During fine-tuning, we utilize the pre-trained Graph Encoder as a molecular representation network and add corresponding output heads to predict a series of molecular properties.

### Encoder

To facilitate the interaction of fine-grained features across different modalities, we use two independent encoders to convert the data of two entirely different modalities into embeddings of the same dimensions for being further processed by Transformer model.

For the SMILES sequence, we follow ChemBERTa [11] to first convert the masked SMILES tokens ${T_{S}}^{M}$ into a sequence of token ids ${ID_{S}}^{M}$, and we expand its vocabulary by conducting a comprehensive analysis of all tokens in our dataset, as detailed in Supplementary D. Then, we calculate their corresponding embeddings $ F_{S}\in \mathbb{R}^{N_{S}\times d}$ by a Transformer model with a series of muti-head attention blocks used in Roberta [37], where $N_{S}$ represents the number of SMILES tokens, and $d$ is the feature dimension.

For the graph, we feed ${V_{G}}^{M}$ and $E_{G}$ into the Graph Encoder. We implement CoMPT [38] as our Graph Encoder, which strengthens the message interactions between nodes and edges through a communicative kernel. After the Graph Encoder processing, we obtain the token embeddings $F_{G}\in \mathbb{R}^{N_{G}\times d}$ for nodes, where $N_{G}$ is the number of atoms, and $d$ is the feature dimension.

### Unified backbone

We design a unified backbone based on Transformer to promote feature interaction between the two modalities. Through the attention mechanism of Transformer, we enable the model to learn the correlation between different input token embeddings across two modalities. After the processing of the two modality-specific encoders, we add trainable parameters $A_{S}\in \mathbb{R}^{N_{S}\times d}$ and $A_{G}\in \mathbb{R}^{N_{G}\times d}$, respectively, to $F_{S}\in \mathbb{R}^{N_{S}\times d}$ and $F_{G}\in \mathbb{R}^{N_{G}\times d}$ and concatenate them. We add learnable parameters just to help the unified backbone to distinguish modalities. Then, the concatenated embeddings $F_{S,G}\in \mathbb{R}^{\left (N_{S}+N_{G}\right )\times d}$ are then fed into the backbone network. Here, we use the Transformer model employed in Roberta [37] as the backbone network with a series multi-head self-attention blocks. The self-attention mechanism is formulated as 


(1) \begin{align*}& \begin{aligned} &\text{SelfAttention}\left(Q,K,V\right)=\text{softmax}\left(\frac{QK^{T}}{\sqrt{d}}\right)V, \end{aligned}\end{align*}

where $Q$, $K$ and $V$ are the projection vectors of $F_{S,G}$, $Q, K, V=F_{S,G}\times W_{Q}, F_{S,G}\times W_{K}, F_{S,G}\times W_{V}$. Through multi-head self-attention mechanism, we can facilitate information interaction between token embeddings both within the same modalities and across different modalities. Finally, the unified backbone outputs the embeddings $F_{S,G}^{\prime }\in \mathbb{R}^{\left (N_{S}+N_{G}\right )\times d}$.

### Decoder

After feature extraction in the backbone, we split the output features $F_{S,G}^{\prime }\in \mathbb{R}^{\left (N_{S}+N_{G}\right )\times d}$ into features $F^{\prime}_{S}\in \mathbb{R}^{N_{S}\times d}$ for SMILES and features $F_{G}^{\prime }\in \mathbb{R}^{N_{G}\times d}$ for graph. $F_{S}^{\prime }$ and $F_{G}^{\prime }$ are used for modality-specific mask reconstruction tasks. Specifically, $F_{S}^{\prime }$ is fed into the SMILES Decoder, which is the LMhead in Roberta [37], to predict the masked token IDs, while $F_{G}^{\prime }$ is inputted into the Graph Decoder, which is a lightweight network GIN [39], after re-masking [40] to reconstruct the masked node features. We calculate the entropy loss $\mathcal{L}_{EN}$ [37] in SMILES reconstruction and the SCE loss $\mathcal{L}_{SCE}$ [40] in graph reconstruction. The overall loss for the entire task is as follows: $\mathcal{L}_{Total}=\mathcal{L}_{EN}+\mathcal{L}_{SCE}$.

### Non-overlapping masking strategy

**Figure 3:**
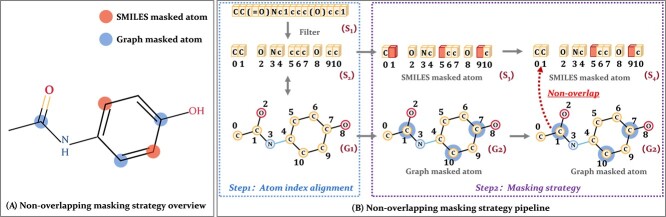
Illustration of non-overlapping masking strategy. (A) Non-overlapping masking strategy: masks in the SMILES sequence and the graph for the same molecule do not overlap. (B) Non-overlapping masking strategy pipeline: first, we establish a correspondence between atom index in both modalities. Then, random masking is applied to the graph, followed by mapping the masked atoms from the graph to the SMILES sequence. Finally, random masking on the SMILES sequence is implemented on the remaining unmasked atoms of the graph.

The non-overlapping masking strategy we propose is illustrated in Fig. 3, which can be divided into two steps, first performing atom index alignment between the two modalities, and then performing non-overlapping masking.

Step 1: Atom index alignment. The SMILES tokens can be categorized into three classes: (1) Atoms, including single-character atoms like C and N, as well as multi-character atoms like Ca and Au, and ions like [Cl-] and [Fe+3]; (2) Chemical bonds, represented by symbols like ‘#’ and ‘=’; (3) Other symbols, such as numbers ‘1’ and ‘2’ indicating the positions of atoms in a ring and parentheses ‘(’ and ‘)’ denoting containing side chains. Given that single bonds are often omitted in SMILES, achieving a one-to-one correspondence between two modalities for chemical bonds is not practical. Therefore, in this paper, we focus on aligning the atom index. Thus, we gather the tokens representing the atoms and assign indexes to them to establish a consistent correspondence between atoms in graph and those in filtered SMILES tokens, as shown in Fig. 3.

Step 2: Masking strategy. We randomly mask atom features on the graph and atom tokens on the SMILES sequence, where the sets of masked atom indexes on them are denoted as $I_{G}$ and $I_{S}$, respectively. To encourage better interaction between the two modalities, we set the overlap ratio between masked atoms in both modalities to 0. Specifically, based on the one-to-one correspondence of atom index, we localize the positions of the masked atoms in graph onto the SMILES sequence. Through operation $P: I_{S}-I_{G} \cap I_{S}$, we avoid masking atoms on the SMILES sequence that are already masked on the graph.

### Fine-tuning

We conduct fine-tuning on the pre-trained Graph Encoder on 14 downstream tasks of predicting molecular properties. Since previous works only utilize a single modality in the downstream tasks, we also take a single modality as input to achieve a fair comparison. We also analyze combining two modalities encodes during fine-tuning; more details can be obtained in Supplementary F. The backbone is not utilized in downstream tasks because it is pre-trained by both modalities and is not suitable for single-modality inputs in downstream tasks.

### Datasets setup

During the pre-training stage, we sample 250 000 unlabeled molecules from ZINC15 [51], which is a comprehensive collection of chemical compounds for drug discovery and computational chemistry research. During the fine-tuning stage, we utilize 14 benchmark datasets from MoleculeNet [52], covering molecular data from various domains, including pharmaceuticals, biology, chemistry and physics. These downstream datasets include 678 binary classification tasks and 19 regression tasks. For more detailed information about benchmark datasets, please refer to Supplementary A.

We partition each benchmark dataset into the train, validation and test sets in an 8:1:1 ratio. For all datasets except QM9, we employ scaffold splitting, reporting the mean and standard deviation of results from three random seeds for each benchmark, and we list seed in Supplementary B. Scaffold splitting is a more challenging and realistic data partitioning method [53]. For the QM9 dataset, we follow the approach used in most prior work [22, 50] for random splitting.

### Baselines and training details

We compare MoleSG with both supervised (training from scratch) baselines and pre-trained baselines. Supervised methods include MPNN [41], DMPNN [42], CMPNN [43], CoMPT [38] and GreSeq [44]. Pre-training methods include N-gram [45], PretrainGNN [46], MGSSL [47], GROVER [7], GraphMVP [20], MolCLR [22], GEM [48], DVMP [21], KANO [50], Mole-Bert [49] and SMICLR [35]. The specific configurations for these competitors can be found in Supplementary C. Additionally, for a fair comparison, we implement new MolCLR and DVMP by replacing the original encoders in them with the same networks we use, which are denoted as $\text{MolCLR}_{\text{CoMPT}}$ and $\text{DVMP}_{\text{MoleSG}}$. We also utilize our non-overlapping masking strategy in $\text{DVMP}_{\text{MoleSG}}$.

We train MoleSG for 90k iterations using the AdamW optimizer with a base learning ratio of 1e-3. We set the mask ratio for graph at 25% and for SMILES at 15%. The details of the mask ratio setting experiments for the two modalities are shown in Section 4.3. We set a maximum of 150 training epochs, with early stopping applied when the validation set’s best value is not improved for more than 20 epochs. We use the AdamW optimizer with a base learning rate of 1e-3 and different warmup factors for different benchmarks. More details of experimental settings can be obtained in Supplementary B.

## Results and discussion

### MoleSG boost the performance of property prediction

In Table 1 and Table 2, the results of MPNN, DMPNN, CMPNN, N-gram, PretrainGNN, MGSSL, GROVER, GraphMVP, MolCLR, GEM and KANO are taken from the paper of KANO [50], while the results of DVMP is obtained from the original text of its original article [21]. As Mole-Bert [49] uses a different data split setting with KANO, we rerun it with the same data split setting as other baselines. Since the number of experimental repetitions of CoMPT [38] is different, we also rerun it using our experimental settings. For two multi-modality methods for SMILES and graph, GraSeq [44] and SMICLR [35], we fully tune them and achieve their best performance for comparison based on their original codes using our experimental settings.

**Table 1:** Performance of different models on eight classification benchmarks in physiology and biophysics. The mean and standard deviation of ROC-AUC (%) from three independent runs are reported (higher values indicate better performance)

Category	Physiology					Biophysics		
Dataset	BBBP	Tox21	ToxCast	SIDER	ClinTox	BACE	MUV	HIV
Molecules	2039	7831	8575	1427	1478	1513	93087	41127
Tasks	1	12	617	27	2	1	17	1
MPNN[41]	91.3$\pm $4.1	80.8$\pm $2.4	69.1$\pm $3.0	59.5$\pm $3.0	87.9$\pm $5.4	81.5$\pm $1.0	75.7$\pm $1.3	77.0$\pm $1.4
DMPNN[42]	91.9$\pm $3.0	75.9$\pm $0.7	63.7$\pm $0.2	57.0$\pm $0.7	90.6$\pm $0.6	85.2$\pm $0.6	78.6$\pm $1.4	77.1$\pm $0.5
CMPNN[43]	92.7$\pm $1.7	80.1$\pm $1.6	70.8$\pm $1.3	61.6$\pm $0.3	89.8$\pm $0.8	86.7$\pm $0.2	79.0$\pm $2.0	78.2$\pm $2.2
CoMPT[38]	96.1$\pm $0.4	84.5$\pm $0.7	72.2$\pm $0.8	66.1$\pm $0.9	97.3$\pm $2.5	94.1$\pm $3.6	82.6$\pm $1.6	86.4$\pm $1.2
GraSeq[44]	92.8$\pm $1.8	78.3$\pm $1.1	70.3$\pm $1.1	65.9$\pm $2.7	82.5$\pm $2.5	89.4$\pm $2.7	72.2$\pm $2.7	83.3$\pm $2.4
N-Gram[45]	91.2$\pm $0.3	76.9$\pm $2.7	-	63.2$\pm $0.5	87.5$\pm $2.7	79.1$\pm $1.3	76.9$\pm $0.7	78.7$\pm $0.4
PretrainGNN[46]	70.8$\pm $1.5	78.7$\pm $0.4	65.7$\pm $0.6	62.7$\pm $0.8	72.6$\pm $1.5	84.5$\pm $0.7	81.3$\pm $2.1	79.9$\pm $0.7
MGSSL[47]	70.5$\pm $1.1	76.4$\pm $0.4	64.1$\pm $0.7	61.8$\pm $0.8	80.7$\pm $2.1	79.7$\pm $0.8	78.7$\pm $1.5	79.5$\pm $1.1
GEM[48]	88.8$\pm $0.4	78.1$\pm $0.4	68.6$\pm $0.2	63.2$\pm $1.5	90.3$\pm $0.7	87.9$\pm $1.1	75.3$\pm $1.5	81.3$\pm $0.3
GROVER[7]	86.8$\pm $2.2	80.3$\pm $2.0	56.8$\pm $3.4	61.2$\pm $2.5	70.3$\pm $13.7	82.4$\pm $3.6	67.3$\pm $1.8	68.2$\pm $1.1
GraphMVP[20]	72.4$\pm $1.6	75.9$\pm $0.5	63.1$\pm $0.4	63.9$\pm $1.2	79.1$\pm $2.8	81.2$\pm $0.9	77.7$\pm $0.6	77.0$\pm $1.2
Mole-Bert[49]	92.9$\pm $1.9	84.5$\pm $3.5	71.5$\pm $0.5	63.4$\pm $2.3	74.7$\pm $10.0	92.6$\pm $1.9	84.7$\pm $1.1	86.2$\pm $1.0
SMICLR[35]	95.7$\pm $2.9	82.5$\pm $2.6	68.7$\pm $1.6	61.3$\pm $2.1	77.4$\pm $21.5	87.4$\pm $3.0	75.2$\pm $3.1	78.1$\pm $3.9
DVMP[21]	77.8$\pm $0.3	79.1$\pm $0.4	-	69.8$\pm $0.6	95.6$\pm $0.7	89.4$\pm $0.8	-	81.4$\pm $0.4
$\text{DVMP}_{\text{MoleSG}}$	80.9$\pm $2.1	84.4$\pm $1.2	73.3$\pm $0.9	66.9$\pm $1.2	98.4$\pm $2.0	93.5$\pm $2.8	80.9$\pm $2.1	87.6$\pm $1.8
MolCLR[22]	73.3$\pm $1.0	74.1$\pm $5.3	65.9$\pm $2.1	61.2$\pm $3.6	89.8$\pm $2.7	82.8$\pm $0.7	78.9$\pm $2.3	77.4$\pm $0.6
$\text{MolCLR}_{\text{CoMPT}}$	97.2$\pm $0.2	82.4$\pm $1.8	72.7$\pm $0.5	57.1$\pm $8.7	77.0$\pm $14.5	85.5$\pm $0.9	75.8$\pm $15.0	81.8$\pm $2.2
KANO[50]	96.0$\pm $1.6	83.7$\pm $1.3	73.2$\pm $1.6	65.2$\pm $0.8	94.4$\pm $0.3	93.1$\pm $2.1	83.7$\pm $2.3	85.1$\pm $2.2
MoleSG	**97.9$\pm $0.3**	**85.0$\pm $1.2**	**74.2$\pm $0.5**	**70.0$\pm $0.2**	**99.1$\pm $0.9**	**95.1$\pm $2.1**	**85.1$\pm $0.8**	**87.7$\pm $1.9**

**Table 2:** Performance of different models on six regression benchmarks in physical chemistry and quantum mechanics. The mean and standard deviation of root mean square error (RMSE) (for ESOL, FreeSolv and Lipophilicity) or mean absolute error (MAE) (for QM7, QM8 and QM9) from three independent runs are reported (lower values indicate better performance)

Category	Physical chemistry			Quantum mechanics		
Dataset	ESOL	FreeSolv	Lipophilicity	QM7	QM8	QM9
Molecules	1128	642	4200	6830	21786	133885
Tasks	1	1	1	1	12	3
MPNN[41]	1.167$\pm $0.043	1.621$\pm $0.952	0.672$\pm $0.051	111.4$\pm $0.9	0.0148$\pm $0.001	0.00522$\pm $0.00003
DMPNN[42]	1.050$\pm $0.008	1.673$\pm $0.082	0.683$\pm $0.016	103.5$\pm $8.6	0.0156$\pm $0.001	0.00514$\pm $0.00001
CMPNN[43]	0.798$\pm $0.112	1.570$\pm $0.442	0.614$\pm $0.029	75.1$\pm $3.1	0.0153$\pm $0.002	0.00405$\pm $0.00002
CoMPT[38]	0.643$\pm $0.051	0.970$\pm $0.207	0.572$\pm $0.058	32.7$\pm $7.4	0.0120$\pm $0.001	0.00353$\pm $0.00067
GraSeq[44]	0.770$\pm $0.035	1.266$\pm $0.131	0.834$\pm $0.009	101.8$\pm $2.7	0.0190$\pm $0.002	0.01890$\pm $0.00010
N-Gram[45]	1.100$\pm $0.030	2.510$\pm $0.191	0.880$\pm $0.121	125.6$\pm $1.5	0.0320$\pm $0.003	0.00964$\pm $0.00031
PretrainGNN[46]	1.100$\pm $0.006	2.764$\pm $0.002	0.739$\pm $0.003	113.2$\pm $0.6	0.0215$\pm $0.001	0.00922$\pm $0.00004
GEM[48]	0.813$\pm $0.028	1.748$\pm $0.114	0.674$\pm $0.022	60.0$\pm $2.7	0.0163$\pm $0.001	0.00562$\pm $0.00007
GROVER[7]	1.423$\pm $0.288	2.947$\pm $0.615	0.823$\pm $0.010	91.3$\pm $1.9	0.0182$\pm $0.001	0.00719$\pm $0.00208
SMICLR[35]	0.883$\pm $0.193	1.345$\pm $0.132	0.861$\pm $0.032	37.5$\pm $7.2	0.0164$\pm $0.001	0.00560$\pm $0.00020
DVMP[21]	0.817$\pm $0.024	1.952$\pm $0.061	0.653$\pm $0.002	74.4$\pm $1.2	0.0171$\pm $0.004	-
$\text{DVMP}_{\text{MoleSG}}$	0.669$\pm $0.114	0.942$\pm $0.110	0.594$\pm $0.018	30.2$\pm $3.0	0.0123$\pm $0.001	0.00323$\pm $0.00006
MolCLR[22]	1.113$\pm $0.023	2.301$\pm $0.247	0.789$\pm $0.009	90.9$\pm $1.7	0.0185$\pm $0.013	0.00480$\pm $0.00003
$\text{MolCLR}_{\text{CoMPT}}$	0.849$\pm $0.062	1.135$\pm $0.163	0.657$\pm $0.012	32.7$\pm $2.8	0.0141$\pm $0.001	0.00350$\pm $0.00000
KANO[50]	0.670$\pm $0.019	1.142$\pm $0.258	0.566$\pm $0.007	56.4$\pm $2.8	0.0123$\pm $0.000	0.00320$\pm $0.00001
MoleSG	**0.599$\pm $0.067**	**0.932$\pm $0.131**	**0.545$\pm $0.014**	**29.6$\pm $2.9**	**0.0117$\pm $0.001**	**0.00313$\pm $0.00006**

Table 1 presents the test results in classification tasks. It can be observed that MoleSG consistently outperforms other methods across all eight datasets, demonstrating its effectiveness. It is worth noting that though the Toxcast dataset benchmark with 617 binary classification tasks is challenging, our method still performs better than the current SOTA method KANO. Complementary information of the two modalities in MoleSG contributes to outstanding results, surpassing methods injecting additional 3D information.

Table 2 shows the test results in regression tasks. We can observe that MoleSG achieves the best scores among both supervised and self-supervised pre-training models, with a relative improvement of 14.4% over KANO across all six regression tasks. MoleSG greatly benefits tasks with limited label information, achieving an 18.4% improvement over KANO on the small dataset FreeSolv, which contains only 642 labeled molecules.

Moreover, it is worth noting that our proposed method still outperforms $\text{MolCLR}_{\text{CoMPT}}$, which is a version of the typical contrastive learning method MolCLR with the same encoder as ours, verifying the superiority of our method. We also compare with another contrastive learning competitor $\text{DVMP}_{\text{MoleSG}}$, which utilizes the same encoders as ours. In addition, both $\text{MolCLR}_{\text{CoMPT}}$ and $\text{DVMP}_{\text{MoleSG}}$ outperform their original counterpart MolCLR and DVMP in most tasks, demonstrating the effectiveness of the corresponding strategies proposed in this paper.

### MoleSG outperforms single-modality pre-training only

To further reveal the superiority of our multi-modality method, we compare our multi-modality pre-training with single-modality pre-training and the results are shown in Table 3 and Table 4. In this experiment, an output head is added to each encoder. ‘SMILES scratch’ and ‘Graph scratch’ represent the two networks trained from scratch. The initial weights of encoders in ‘SMILES pre-train’and ‘Graph pre-train’ are obtained from single-modality pre-training using the same MoleSG framework while blocking the other modality. The initial weights of encoders in ‘Ours SMILES’ and ‘Ours graph’ are obtained from the corresponding encoders of the multi-modality pre-trained MoleSG. From these results, we can observe that our proposed method achieves the best performance on all downstream tasks. Moreover, it is worth noting that single modality pre-training may cause performance degradation. However, by fully leveraging the complementary information among different modalities, our method can improve performance on all downstream tasks, showing more potential for practical applications.

**Table 3:** Comparison of our approach with two single-modality pre-training approaches on classification tasks. The mean and standard deviation of ROC-AUC (%) over three independent runs are reported (higher values indicate better performance)

	BBBP	Tox21	ToxCast	SIDER	Clintox	BACE	MUV	HIV
SMILES scratch	63.6$\pm $4.3	75.5$\pm $0.5	64.2$\pm $2.5	54.0$\pm $2.4	88.1$\pm $6.3	79.2$\pm $6.6	63.6$\pm $4.3	72.7$\pm $3.5
SMILES pre-train	61.5$\pm $4.9	77.6$\pm $2.5	66.8$\pm $0.9	55.0$\pm $3.1	93.3$\pm $2.8	83.8$\pm $0.9	61.5$\pm $4.9	75.1$\pm $2.5
Ours SMILES	**65.3$\pm $3.1**	**77.9$\pm $2.5**	**67.0$\pm $0.9**	**59.6$\pm $3.8**	**94.3$\pm $2.0**	**85.3$\pm $1.1**	**65.3$\pm $3.1**	**77.3$\pm $0.7**
Graph scratch	96.1$\pm $0.4	84.5$\pm $0.7	72.2$\pm $0.8	66.1$\pm $0.9	97.3$\pm $2.5	94.1$\pm $3.6	82.6$\pm $1.6	86.4$\pm $1.2
Graph pre-train	96.8$\pm $1.8	84.2$\pm $0.1	72.6$\pm $1.0	66.7$\pm $2.2	98.0$\pm $0.9	94.9$\pm $2.3	82.2$\pm $1.4	85.9$\pm $2.5
Ours graph	**97.9$\pm $0.3**	**85.0$\pm $1.2**	**74.2$\pm $0.5**	**70.0$\pm $0.2**	**99.1$\pm $0.9**	**95.1$\pm $2.1**	**85.1$\pm $0.8**	**87.7$\pm $1.9**

**Table 4:** Comparison of our approach with two single-modality pre-training approaches on regression tasks. The mean and standard deviation of RMSE or MAE over three independent runs are reported (lower values indicate better performance)

	ESOL	Freesolv	Lipophilicity	QM7	QM8	QM9
SMILES scratch	0.946$\pm $0.226	2.581$\pm $0.286	1.028$\pm $0.030	160.2$\pm $6.8	0.0146$\pm $0.001	0.01017$\pm $0.00045
SMILES pre-train	1.030$\pm $0.336	1.942$\pm $0.450	1.034$\pm $0.015	159.3$\pm $5.7	0.0141$\pm $0.001	0.01080$\pm $0.00010
Ours SMILES	**0.873$\pm $0.172**	**1.889$\pm $0.590**	**0.964$\pm $0.036**	**155.7$\pm $3.9**	**0.0139$\pm $0.001**	**0.00973$\pm $0.00059**
Graph scratch	0.643$\pm $0.051	0.970$\pm $0.207	0.572$\pm $0.058	32.7$\pm $7.4	0.0120$\pm $0.001	0.00353$\pm $0.00067
Graph pre-train	0.635$\pm $0.104	0.939$\pm $0.225	0.585$\pm $0.031	32.3$\pm $1.6	0.0118$\pm $0.001	0.00323$\pm $0.00012
Ours graph	**0.599$\pm $0.067**	**0.932$\pm $0.131**	**0.545$\pm $0.014**	**29.6$\pm $2.9**	**0.0117$\pm $0.001**	**0.00313$\pm $0.00006**

**Figure 4:**
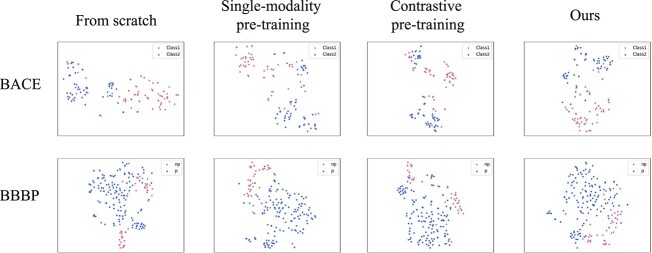
T-SNE visualization of feature separation of four methods on BACE and BBBP benchmark datasets.

We present visualization results of our method’s feature extraction capability in Fig. 4, which illustrates the strong feature discriminative ability of MoleSG in the classification tasks BBBP and BACE. We compare our proposed model with models trained from scratch (without pre-training), from single-modality pre-training (i.e. graph pre-training), and from contrastive pre-training ($\text{DVMP}_{\text{MoleSG}}$). During fine-tuning, these competitors all utilize the Graph Encoder. From Fig. 4, we can observe the superior feature discrimination of our approach compared with single-modality pre-training and contrastive pre-training. Learning efficiency analysis can be seen in Supplementary G.

### Mask ratio setup

**Figure 5:**
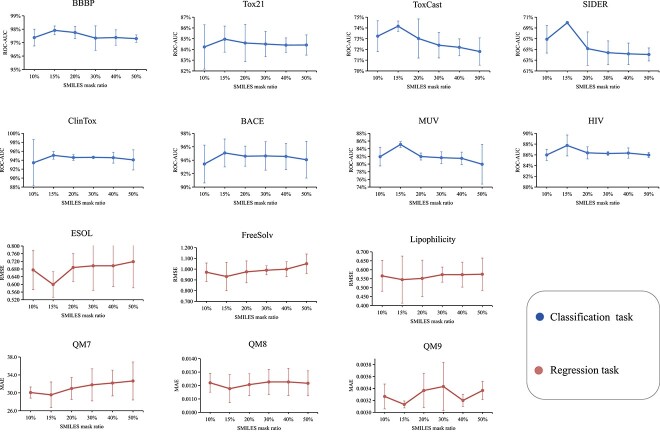
The impact of different mask ratios on downstream task performance on SMILES. The results are reported as mean and standard deviation values on three independent runs.

**Figure 6:**
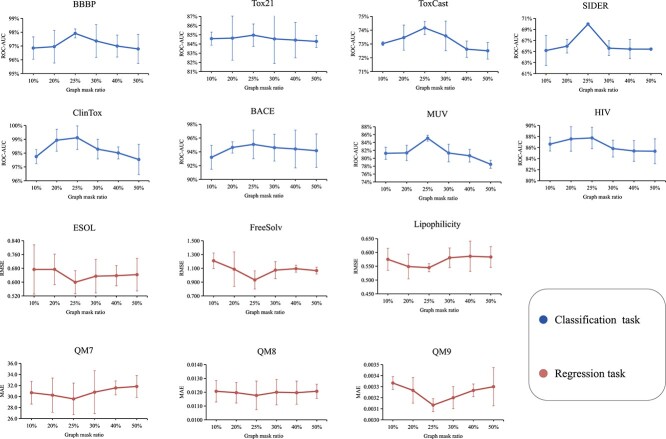
The impact of different mask ratios on downstream task performance on graph. The results are reported as mean and standard deviation values on three independent runs.

To determine the mask ratio for graph and SMILES modalities, we use a controlled variable approach. We adjust the graph mask ratio while keeping the SMILES mask ratio constant, and vice versa. We conduct experiments across all benchmarks, and the experimental results for the SMILES mask ratio and graph mask ratio are shown in Fig. 5 and Fig. 6, respectively. We observe that a SMILES mask ratio of 15% and a graph mask ratio of 25% are suitable for our purposes. (In classification tasks, a higher ROC-AUC(%) value indicates better performance, while in regression tasks, lower RMSE and MAE values are desirable.)

### Ablation experiments

#### Overlap versus non-overlap

**Figure 7:**
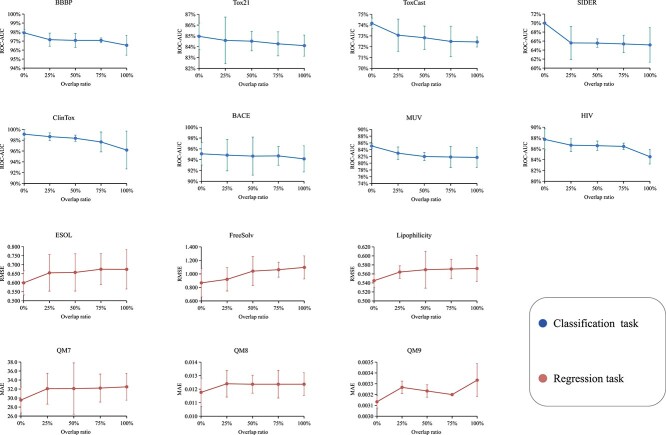
The impact of different overlap ratios on downstream task performance. The results are reported as mean and standard deviation values on three independent runs.

To validate whether our non-overlapping masking strategy benefits pre-training, we conduct experiments on different overlap ratios on all downstream tasks. We define overlap ratio as a metric measuring the proportion of jointly masked atoms in both modality inputs. We conduct experiments at overlap ratios at 0%, 25%, 50%, 75% and 100% across all benchmarks, where our non-overlapping masking strategy is equivalent to setting the overlap ratio to 0. The experimental results shown in Fig. 7 indicate that the performance on downstream tasks is the best when the overlap ratio is 0.

#### With versus without backbone

As analyzed in Section 3.6, fine-tuning both the encoder and backbone may cause suboptimal performance due to inconsistent distributions. Therefore, we conduct an experiment to validate it. Specifically, Table 3 and Table 4 show that the performance of Graph Encoder is better than SMILES Encoder. Therefore, we only consider two combinations in this section. The former is fine-tuning a single Graph Encoder, and the other is fine-tuning both the Graph Encoder and the backbone. We perform experiments on all benchmarks, and the results are shown in Table 5 and Table 6. The results show that using only the Graph Encoder achieves higher performance in all tasks.

**Table 5:** Comparison of results on classification tasks with and without the backbone network. The mean and standard deviation of ROC-AUC (%) from three independent runs are reported (higher values indicate better performance)

	BBBP	Tox21	ToxCast	SIDER	ClinTox	BACE	MUV	HIV
Graph Encoder+backbone	97.2$\pm $0.6	84.8$\pm $1.8	73.6$\pm $0.9	65.6$\pm $0.4	98.8$\pm $0.6	89.7$\pm $5.2	81.9$\pm $1.9	85.8$\pm $1.4
Graph Encoder	**97.9$\pm $0.3**	**85.0$\pm $1.2**	**74.2$\pm $0.5**	**70.0$\pm $0.2**	**99.1$\pm $0.9**	**95.1$\pm $2.1**	**85.1$\pm $0.8**	**87.7$\pm $1.9**

**Table 6:** Comparison of results on regression tasks with and without the backbone network. The mean and standard deviation of RMSE (or MAE) from three independent runs are reported (lower values indicate better performance)

	ESOL	FreeSolv	Lipophilicity	QM7	QM8	QM9
Graph Encoder+backbone	0.661$\pm $0.011	0.988$\pm $0.250	0.560$\pm $0.017	31.9$\pm $3.8	0.0119$\pm $0.001	0.00353$\pm $0.00015
Graph Encoder	**0.599$\pm $0.067**	**0.932$\pm $0.131**	**0.545$\pm $0.014**	**29.6$\pm $2.9**	**0.0117$\pm $0.001**	**0.00313$\pm $0.00006**

## Conclusion

In this study, we address the challenges of learning fine-grained information from two complementary modalities: SMILES and graph. To better capture rich molecular features from the interaction between these two modalities, we design a simple and efficient multi-modality pre-training framework called MoleSG, which utilizes a unified feature processing network to fuse both modalities. In addition, we propose a non-overlapping masking strategy to facilitate information exchange between the two modalities. Extensive experiments on 14 downstream tasks show that our method achieves new SOTA performance. Our non-overlapping masking strategy has the potential to be used in other masked reconstruction-based multi-modality pre-training studies.

There are two potential directions for future work. (1) Our multi-modality pre-training method can be utilized in the protein representation learning, because proteins also have both sequence and graph representations. (2) Our non-overlapping masking strategies can be extended to other joint pre-training studies of multiple-modality data.

**Key Points**

- MoleSG is a novel molecular pre-training framework that utilizes the complementary information of SMILES and graph representations, resulting in improved performance.
- To achieve information interactions in fine-grain, we design a unified network for handling two distinct modalities, allowing for fine-grained interaction between SMILES and graph representations and achieving better representation learning.
- To better learn complementary information across two modalities, we introduce an innovative non-overlapping masking strategy to encourage the model to reconstruct the masked part of one modality using the direct information of the corresponding part of the other modality, which strengthens the interactions between the two modalities.
- MoleSG achieves SOTA performance in a series of molecular property prediction tasks, and a detailed ablation study demonstrates that our proposed multi-modality method outperforms single-modality pre-training and the masking strategy promotes performance.

## Supplementary Material

Supplementary_bbae256

## Data Availability

Data and codes in our experiments are released in https://github.com/ShenAoAO/MoleSG.

## Author Biographies

**Ao Shen** is a PhD candidate at Fudan University. Her research interests are AI4Science and drug screening.

**Mingzhi Yuan** is a PhD candidate at Fudan University. His research interests are 3D computer vision, machine learning, and AI4Science.

**Yingfan Ma** is a Master degree candidate at Fudan University. Her research interests are computer vision and AI4Science.

**Jie Du** is a Master degree candidate at Fudan University. His research interests are artificial intelligence and molecular design.

**Manning Wang** is a professor at Fudan University. His research interests are 3D computer vision, deep learning, and AI4Science.
