# Supplementary material for: Complementary multi-modality molecular self-supervised learning via non-overlapping masking for property prediction
Source: Brief Bioinform. 2024 May 27;25(4):bbae256. doi: 10.1093/bib/bbae256 (PMC11129775; doi:10.1093/bib/bbae256)
Supplement: Supplementary_bbae256 [file supplementary_bbae256.pdf]

Supplementary

A Downstream data supplements

The 14 downstream task datasets are sourced from MoleculeNet [34] and can be categorized into physiology (BBBP, Tox21, ToxCast, SIDER, ClinTox), biophysics (BACE, MUV, HIV), physical chemistry (ESOL, FreeSolv, Lipophilicity), and quantum mechanics (QM7, QM8, QM9). All downstream tasks are also small molecule data tasks, with the number of atoms not exceeding 140. Our pre-training data set is the small molecule database ZINC15, with a maximum number of atoms of 26.

Physiology focuses on macroscopic life systems, while biophysics employs physical methods to study biological phenomena. Physical chemistry analyzes the principles and laws governing the chemical behaviour of matter systems from a physical perspective, while quantum mechanics does the same for the chemical behaviour of matter systems from a physics perspective.

Table 1 provides detailed information on these 14 datasets, including task types, evaluation metrics, molecular categories, data size, and split types. As shown in Table 1, we employed scaffold splitting for all benchmarks except QM9. Scaffold splitting segregates molecules with different two-dimensional structural frameworks into distinct subsets, offering a more challenging yet practical setup where test molecules can differ structurally from the training set. For QM9, we used random splitting based on previous research. Our evaluation metrics for regression tasks were RMSE and MAE.

Table 1. The detailed information of all the benchmarks for molecular property predictions used in this work. The benchmarks contain 8 graph classification datasets and 6 graph regression datasets.

<table>
<tr><th>Dataset</th><th>Task Type</th><th>Metric</th><th>Category</th><th>Tasks</th><th>Compounds</th><th>Split</th></tr>
<tr><td>BBBP</td><td rowspan="8">Classification</td><td rowspan="8">ROC-AUC</td><td rowspan="5">Physiology</td><td>1</td><td>2039</td><td rowspan="13">scaffold split</td></tr>
<tr><td>Tox21</td><td>12</td><td>7831</td></tr>
<tr><td>ToxCast</td><td>617</td><td>8575</td></tr>
<tr><td>SIDER</td><td>27</td><td>1427</td></tr>
<tr><td>ClinTox</td><td>2</td><td>1478</td></tr>
<tr><td>BACE</td><td rowspan="3">Biophysics</td><td>1</td><td>1513</td></tr>
<tr><td>MUV</td><td>17</td><td>93087</td></tr>
<tr><td>HIV</td><td>1</td><td>41127</td></tr>
<tr><td>ESOL</td><td rowspan="6">Regression</td><td rowspan="3">RMSE</td><td rowspan="3">Physical chemistry</td><td>1</td><td>1128</td></tr>
<tr><td>FreeSolv</td><td>1</td><td>642</td></tr>
<tr><td>Lipophilicity</td><td>1</td><td>4200</td></tr>
<tr><td>QM7</td><td rowspan="3">MAE</td><td rowspan="3">Quantum mechanics</td><td>1</td><td>6830</td></tr>
<tr><td>QM8</td><td>12</td><td>21786</td></tr>
<tr><td>QM9</td><td>3</td><td>133885</td><td>random split</td></tr>
</table>

The dataset details are as follows:

1. BBBP [1] comprises information concerning whether a compound exhibits the capability to traverse the blood-brain barrier.

2. Tox21 [2] is a publicly accessible database designed to assess the toxicity profiles of various

compounds, notably in the 2014 Tox21 Data Challenge.
3. ToxCast [3] houses an extensive array of toxicity labels for thousands of compounds, derived from high-throughput screening tests on a vast chemical library.
4. SIDER [4] archives information on commercially available medications, complete with details on their associated adverse drug reactions, often referred to as the Side Effect Resource.
5. ClinTox [5] conducts a comparative analysis between drugs that have received FDA approval and those that have been eliminated during clinical trials due to safety concerns.
6. BACE [6] serves as a repository for compounds identified in recent years as potential inhibitors of human β-secretase 1 (BACE-1).
7. MUV [7] represents a refined subset of the PubChem BioAssay, specifically tailored for the validation of virtual screening techniques through advanced nearest neighbor analysis.
8. HIV [8] provides experimental data on the inhibitory capabilities of over 40,000 molecules against HIV replication.
9. ESOL [9] is a compact dataset documenting compound solubility.
10. FreeSolv [10] is derived from the Free Solvation Database, containing hydration-free energy data for small molecules in water from experimental and alchemical calculations.
11. Lipophilicity [11] is sourced from the ChEMBL database and contains experimental octanol-water partition coefficient results, reflecting molecule solubility.
12. QM7 [12] is a subset of GDB-13, providing molecular spatial structure information and stable, synthetically obtainable electronic properties such as HOMO, LUMO, and atomization energy, determined using ab-initio density function theory (DFT).
13. QM8 [13] employs various quantum mechanics methods to compute electronic spectra and excited state energies for small molecules.
14. QM9[14] offers extensive data on the geometry, energy, electronic, and thermodynamic properties of small molecules calculated via DFT.

B Experimental settings

Training settings: We train MoleSG for 90k iterations using the AdamW optimizer with a base learning ratio of 1e-3 and a warmup factor of 0.1 for the first 30 epochs. We set the masking ratio for the graph at 25% and for SMILES at 15%. We set the batch size to 32.

Fine-tuning settings: We use the AdamW optimizer with a base learning rate of 1e-3 and different warmup factors for the first 30 epochs. We set a maximum of 150 training epochs, with early stopping applied when the validation dataset's best value is not improved for more than 20 epochs. As shown in Table 2, different downstream tasks adopt targeted model parameters.

Model hyperparameters:

a. Graph Encoder: The atom feature dimension is 116 and the edge feature dimension is 13. The dimension of the output feature is 256. The number of attention heads is 8, and the number of hidden layers is 8.

b. Graph Decoder: The input dimension is 256 and the output dimension is 116. The decoder is a GIN layer.

c. SMILES Encoder, Unified backbone and SMILES Decoder: The vocabulary size is 700, the number of attention heads is 2, and the number of hidden layers is 2. The dimension of the output feature is 256.

Table 2. Training parameters of different downstream tasks.

Dataset	Warmup factor	Batch size	Loss function
BBBP	0.2	32	torch.nn.BCEWithLogitsLoss()
Tox21	0.1	32	torch.nn.BCEWithLogitsLoss()
ToxCast	0.1	32	torch.nn.BCEWithLogitsLoss()
SIDER	0.2	32	torch.nn.BCEWithLogitsLoss()
ClinTox	0.15	32	torch.nn.BCEWithLogitsLoss()
BACE	0.1	32	torch.nn.BCEWithLogitsLoss()
MUV	0.1	32	torch.nn.BCEWithLogitsLoss()
HIV	0.1	24	torch.nn.BCEWithLogitsLoss()
ESOL	0.1	32	torch.nn.MSELoss()
FreeSolv	0.15	32	torch.nn.MSELoss()
Lipophilicity	0.15	32	torch.nn.MSELoss()
QM7	10	32	torch.nn.L1Loss()
QM8	0.1	32	torch.nn.L1Loss()
QM9	10	32	torch.nn.L1Loss()

Random seeds: As shown in Table 3, we list the random number seeds used in each downstream task, which are not only used for data partitioning but also for the rest of the experiments involving random number seeds.

Table 3. Random seeds in 14 downstream tasks.

Dataset	Seeds
BBBP	2,43,51
Tox21	26,43,45
ToxCast	5,50,84
SIDER	257,277,395
ClinTox	58,62,92
BACE	19,29,81
MUV	22,70,76
HIV	51,56,66
ESOL	55,77,85
FreeSolv	29,45,68
Lipophilicity	27,77,81
QM7	9,36,73
QM8	47,69,73
QM9	4,13,16

C Competitors

To verify MolSG's effectiveness, we conduct a thorough performance evaluation, comparing it with supervised and self-supervised learning competitors.

Competitors overview: Supervised competitors contain MPNN [15], DMPNN [16], CMPNN [17], CoMPT[18], and GraSeq [29], which are specifically designed for molecules. GraSeq [29] uses a complementary combination of graph neural networks and recurrent neural networks to model two types of molecular inputs. In our evaluation of predictive-based self-supervised learning, we consider several pre-training models. For instance, N-gram [19] assembles node embeddings in short walks and utilizes Random Forest or XGBoost for predicting molecular properties. PretrainGNN [20] and GROVER [21] incorporate both node-level and graph-level knowledge into pretext tasks. MGSSL [22] employs a pretext task involving motif generation, while GEM [23] focuses on geometry-level self-supervised learning strategies for molecular geometry knowledge acquisition. In the realm of contrastive-based models, we include GraphMVP [24], which integrates 3D geometric information into graph self-supervised learning. Additionally, MolCLR [25] applies general graph augmentation methods to molecules. To ensure a fair comparison, we substitute the original GCN and GIN backbones in MolCLR with the CoMPT backbone, resulting in an additional baseline referred to as $MolCLR_{CoMPT}$ for a comprehensive comparative analysis with our method. DVMP [26] employs a contrastive self-supervised learning approach to obtain knowledge from the same molecule. It extracts SMILES sequence information encoded by Transformers and graph information encoded by Graph Neural Networks (GNNs). For a fair comparison, we replace the feature extraction networks for both SMILES and graphs in DVMP with the same networks used in MoleSG, and the result is shown as $DVMP_{MoleSG}$. We also adopt the same mask strategy as used in our approach. Mole-Bert [27] encodes atoms into meaningful discrete values and design a masked atom model for pre-training. KANO [28] is a molecular contrastive learning method enhanced with knowledge graphs and functional prompts. SMICLR [30] proposes a contrastive learning pre-training method that integrates molecular SMILES and graph modalities.

Competitors experimental setup: In this paper, we compare MoleSG with 16 baseline methods, including MPNN[15], DMPNN[16], CMPNN[17], CoMPT[18], N-gram[19], PretrainGNN[20], MGSSL[22], GROVER[21], GraphMVP[24], MolCLR[25], GEM[23], DVMP[26], KANO[28], ,Mole-Bert [27], GraSeq [29] and SMICLR [30]. The results of MPNN, DMPNN, CMPNN, N-gram, PretrainGNN, MGSSL, GROVER, GraphMVP, MolCLR, GEM, and KANO are taken from the paper of KANO, while the results of DVMP is obtained from the original text of these two articles. As Mole-Bert uses a different data split setting with KANO, we rerun it with the same data split setting as other baselines. Since the number of experimental repetitions of CoMPT is different, we also rerun it using our experimental settings. For two multi-modality methods for SMILES and graph, GraSeq [29] and SMICLR [30], we fully tune them and achieve their best performance for comparison based on their original codes using our experimental settings.

D Token vocabulary

A Simplified Molecular Input Line Entry System (SMILES) is a linear notation used to represent molecules simply and compactly, categorizing their components into three types: atoms, bonds, and other representations encoding ring closures in the graph. An example of a molecule represented in SMILES notation is shown in Figure 1, where the SMILES representation for a molecule with the structure c1cc(F)ccc1Cl is provided alongside its 3D molecular structure. In essence, letters such as C, Cl, and F generally represent atoms, while symbols like -, =, and # represent chemical bonds, and numbers denote adjacent atoms in ring-closing parts of the molecule. However, it's worth noting that the SMILES system is not a perfect one-to-one mapping between SMILES sequences and molecular structures. For instance, a molecule can have multiple equivalent SMILES representations, such as CCO, OCC, and C(O)C. To address this issue and provide a one-to-one mapping between SMILES and molecules, various standardization algorithms have been developed to ensure uniqueness in representing each molecular structure. In this paper, all SMILES representations used are standardized.

Below, we will list all the elements found in the SMILES data, along with their corresponding token IDs. Knowing all the elements indicated by these IDs will facilitate the extraction of atomic representations for our non-overlapping masking strategy.

Token vocabulary:'[13cH]': 279, '[N-3]': 294, '[NH3+2]': 510, '[nH]': 40, '[O+]': 73, '[Br]': 218, '[Th+4]': 534, '[Mn-2]': 682, '[S-2]': 201, '[123I-]': 368, '[NH-]': 161, '[Mg]': 77, '[Nd+]': 412, '[13CH3]': 260, '[Sb]': 200, '[BrH+]': 479, '[35S]': 497, '[Ca-2]': 624, '[YH]': 372, '[Si@]': 268, '[Tl+]': 304, '[Mn+2]': 190, '[2NH]': 588, '[Li-]': 538, 's': 42, '[Se-]': 321, '[3H]': 257, '[SH5]': 504, '[18F-]': 340, '[HgH]': 649, '[BH2]': 442, '[unused10]': 10, '[Ru]': 117, '[Os+4]': 306, '[Co+]': 500, '[11c]': 548, '[Mo-]': 641, '[Si+4]': 245, '[PH2]': 164, '[N+]': 41, ')': 18, '[Cu+]': 131, '[Ra]': 658, '5': 43, '[c+]': 314, 'b': 410, '[N@@H+]': 486, '[Ni-3]': 634, '[NH4]': 594, '[Fm]': 415, '[SH]': 139, '[AlH2]': 377, '[SnH4]': 342, '[c]': 466, '[BaH]': 531, '[unused3]': 3, '[11CH3]': 284, '[13C]': 228, '[Nd+3]': 277, '[18OH2]': 557, '[C@@]': 56, '[Cs+]': 68, '[Si+2]': 567, '[201Tl]': 595, '#': 38, '[Si]': 47, '[Mo+2]': 470, '[13N]': 586, '[C@@H]': 35, '[AlH4-]': 103, '[Er]': 424, '[pH+]': 666, '[Rh+2]': 199, '[PH3]': 329, '[Zr-2]': 501, '[OH-]': 59, '[Zn-4]': 621, '(': 17, 'Br': 37, '[Hg+2]': 186, '[PH4]': 452, '[V+5]': 251, '[In]': 192, '[SH2]': 324, '[Ir+2]': 438, '[15NH2]': 336, '[9CH]': 563, '[As+]': 443, '[Ba]': 207, '-': 31, '[C@H]': 33, '[Ga+]': 429, '[123I]': 313, '[Mo]': 154, '[15OH2]': 587, '[CH]': 83, '[Co+2]': 182, '%12': 202, '[Hg+]': 283, '[PbH2+2]': 647, '[Al-]': 225, '[V]': 223, '[Tl+2]': 518, '[Fe]': 93, '[SiH-]': 673, '[211At]': 509, '[AsH]': 474, '[CH2-]': 157, '[Sn+2]': 168, '[se+]': 439, '[Zn]': 87, '[Ru-4]': 620, '[o+]': 215, '[SeH+]': 434, '%19': 388, '[Cu-]': 224, '[IH2+]': 330, '[Ge]': 209, '[Mo+4]': 382, '[C+4]': 393, '[153Sm]': 561, '[Ti+2]': 269, '[Cd+2]': 309, '[Be+2]': 392, 'p': 206, '[B+2]': 506, '[AsH4]': 689, '[Hf]': 285, '[Li+]': 69, '[Cu-4]': 602, '[14CH2]': 555, '[Sn-]': 669, '[SbH6+3]': 644, '[Pd-2]': 273, '[Sm+3]': 325, '[PH-]': 459, '[GeH2]': 467, '[Dy]': 473, '[SbH]': 487, '[13CH]': 423, '[VH]': 505, '[PH]': 115, '[SnH]': 150, '[Na-]': 454, '[13c]': 227, '[Pa]': 651, '[Ni-4]': 639, '~': 30, '[Cs]': 70, '[Ni+2]': 180, '[Rb+]': 315, '[No]': 665, '[Ni-2]': 631, '[Co-4]': 611, '[Ac-]': 558, '[Ag]': 123, '[SH-]': 173, 'F': 27, '[W]': 149, '[Cd]': 289, '[Yb]': 335, '[TeH]': 343, '[Mn+3]': 320, '[Nd]': 359, '%17': 370, 'o': 44, '[2H]': 64, '[Zr+]': 413, '[Hf+3]': 420, '[In+]': 477, '%13': 217, '[60Co]': 488, '[TlH]': 650, '[SEP]': 13, '[NH2+]': 116, '[Ga-3]': 625, '[Ca-4]': 610, '[Dy+3]': 418, '[Y]': 287, '[unused1]': 1, '[Bi+5]': 571, '%16': 355, '[P-3]': 577, '[Te]': 226, '[IH4]': 687, '[Ac]': 130, '[Cr+3]': 211, '[Sr+2]': 246, '[Ag+]': 118, '[14c]': 369, '[U+2]': 396, '[Er+3]': 367, '[Cr-]': 514, '8': 98, '[Sn+4]': 282, '[SH+]': 236, '%21': 398, '[15N]': 404, '[Cn]': 590, '[UNK]': 11, '[V+]': 608, '[W+2]':

601, '[NH+3]': 536, '[Fe-]': 683, '[Ir-3]': 656, '[Pt]': 109, '[Si-]': 250, '[CaH2]': 346, '[Ti+5]': 496, '[Au]': 212, '[B+]': 305, '[Yb+3]': 187, '[Ca+2]': 122, '[Ag+3]': 449, '%18': 379, '[Sm]': 255, '[TeH3]': 447, '[Mn+6]': 568, '[B-2]': 633, '[SiH3]': 151, '*': 256, '[125I]': 363, '[CH3+]': 317, '[SnH2]': 371, 'I': 48, '[unused4]': 4, '[S@+]': 220, '[SiH4+]': 556, '[AlH3-3]': 640, '[TlH2]': 664, '[67Ga+3]': 584, '[cH-]': 135, '[7NaH]': 539, '[OH+]': 261, '[Pb+2]': 174, '[Mo-3]': 604, '[CH-]': 188, '[Ag+2]': 318, '[Ca]': 128, '[Cu-2]': 276, '[ZrH2]': 569, '[Ba+]': 475, '[SnH2+2]': 646, '[n+]': 79, '[S@]': 108, '[SeH]': 249, '[Ni+]': 446, '[AsH3]': 490, '[Fe+3]': 152, '[NH2]': 258, '%10': 156, '[64Cu]': 519, '[1H]': 522, '[NH+]': 121, '[Rh+]': 292, '[Si@@H]': 344, '[Tl]': 347, '[PH+]': 143, '[C-4]': 358, '[Cm]': 430, '[Ir-]': 391, '[Cf]': 444, '[Cl-]': 57, '[Cr+]': 471, '[U-5]': 606, '[Pd-3]': 600, '[124I-]': 578, '[FH+]': 618, '[Pr]': 286, '[GaH3]': 605, '[BH]': 384, '[Cr]': 105, '[S+4]': 311, '[Mg+]': 147, '[249Cf]': 642, '[Se]': 126, '[U+6]': 433, '[Ni+3]': 516, '[Rh-3]': 216, '[IH-]': 432, 'c': 15, '[(1R)-1-methylpropyl]': 597, '[I-]': 84, '[Cl+3]': 134, '[Sb+3]': 297, '[PH5]': 354, '[68Ga]': 458, '[Hg-2]': 627, '[Zr]': 195, '[Zn+2]': 114, '[cH+]': 319, '[15NH]': 395, '[Mo+6]': 528, '[Zr+2]': 189, '[Pd-]': 308, '[Nb+3]': 581, '[32P]': 435, '[TlH2+]': 643, '[AlH3-]': 630, '[Zn-3]': 622, '[S@@+]': 234, '[RuH2]': 394, '[Ti+3]': 198, '[19F]': 378, '[18O]': 451, '[Pt-]': 437, '[AsH+]': 529, '[Fe+4]': 532, '[Ar]': 222, '[unused9]': 9, '[P+]': 91, '%11': 170, '[13CH2]': 265, '[se]': 142, '[Si@@]': 267, '[Zr+3]': 291, '[Ta+5]': 348, '[11C]': 422, '[H]': 63, '[KH]': 193, '[Rb]': 386, '[Ce+2]': 461, '[Sn+3]': 365, '[IH+]': 326, '[PtH+2]': 541, '[Pt-4]': 617, '[Mn]': 100, '[Cu-5]': 619, '</s>': 592, '[131I]': 503, '[Nb+4]': 580, '[Br+2]': 238, '[Se+]': 575, '[Co]': 162, '[AsH4+]': 489, '[Co+3]': 310, '[Ru+2]': 300, '[SiH4]': 231, '[S-]': 112, '[Ho+3]': 457, '[Re+]': 612, '[Si-2]': 645, '[(2S)-butan-2-yl]': 598, '[PAD]': 0, '[W+4]': 421, '[Cu+3]': 469, '[asH]': 674, '[Ru-]': 527, '[WH]': 690, '[Al+2]': 402, '[Co-3]': 660, '[N@@]': 242, '[N-]': 61, '[Tl+3]': 352, '[K]': 52, '[Eu+3]': 323, 'Cl': 28, '[Al+3]': 110, '[Be]': 385, '[IH]': 361, '[CuH2-]': 579, '[Sn]': 80, '[n-]': 159, '[229Th]': 533, '[HeH]': 551, '[Zr+4]': 176, '[Sb+2]': 455, '[IH2]': 686, '[Ir-4]': 637, '[V+2]': 316, '[H+]': 140, '[Ag-]': 259, '[Gd+3]': 264, '[CH2+]': 155, '[SH2+]': 331, '[La+3]': 240, '[Nb]': 366, '[Na]': 49, '[pH]': 387, '[PH2+]': 414, '[nH+]': 102, '[In+2]': 484, '[unused5]': 5, '[Ir]': 178, '[At]': 576, '[13C@@H]': 464, '%14': 298, '[Ir+4]': 583, '[MgH2]': 629, '[Re]': 253, '[Zn+]': 136, '[31P]': 495, '[Rh-]': 513, '[C-2]': 570, '[Al]': 90, '[N@H+]': 416, '[Gd]': 339, '[b-]': 636, '[NaH]': 71, '[Rh+3]': 235, '[Ta]': 303, '[AlH4]': 95, '[SbH2]': 448, '[CuH2]': 494, '[Sc+2]': 552, '[PdH2]': 307, '[15n]': 401, '[OH3+]': 328, '[p+]': 662, '[I+]': 163, '[Nb+5]': 312, '[Br-]': 85, 'P': 45, '[O-2]': 104, 'N': 23, '[I+3]': 120, '[SiH2]': 127, '[F]': 427, '[BH2-]': 502, '[Ni-]': 545, '[IH+3]': 677, '[Cu+2]': 99, '[PH4+]': 230, '[Al+]': 247, '<s>': 591, '[Bi+]': 540, '[ArH]': 499, '[15CH]': 574, '[Xe]': 390, '[Os-3]': 653, '[BiH2]': 685, '[BrH2+]': 628, '[10B]': 468, '[Pb+4]': 210, '[14cH]': 480, '[NiH2]': 566, '%15': 327, 'S': 34, 'B': 54, '[P@]': 181, '[ghi]': 596, '[W+]': 481, '2': 21, '[Tl-3]': 613, '[Ti]': 113, '[Ni]': 96, '[Cd+]': 521, '[Hf+2]': 295, '[Tb+3]': 403, '[9CH3]': 564, '[P@H]': 537, '[B+3]': 275, '[AlH2-]': 196, '[NH]': 153, '[MASK]': 14, '[Ce+3]': 194, '[Lu]': 337, '[V-]': 615, '[Zn-2]': 609, '[S+]': 119, '\\': 60, '[Randic connectivity]': 593, '[Eu]': 364, '[Pd-4]': 638, '[Am]': 670, '[C+]': 146, '[BH3-]': 92, '[Tb]': 380, '[Cu-3]': 623, '[Sn+]': 270, '[Ga-]': 676, '[SH3+]': 341, '[Tc]': 520, '[Ir-2]': 678, '[Fe+]': 655, '[N@@+]': 356, '[Y+3]': 252, '[P]': 53, '[PbH]': 659, '[Ba+2]': 169, '[Na-2]': 493, '[Si@H]': 204, '[BH4-]': 74, '%20': 389, '[NH2-]': 208, '[Y-]': 565, '[I]': 144, '[SnH3]': 274, '[GeH]': 349, '[Te+]': 667, '[P+3]': 185, '[As+3]': 535, '[O]': 137, '[N@]': 244, '[IrH]': 511, '[Pb]': 248, '[Xe+]': 546, '[SeH-]': 436, '[unused2]': 2, '[Os-2]': 175, '[Au+3]': 357, '[14C@H]': 508, '[Fe+6]': 465, 'C': 16, '.': 24, '6': 58, '[Pt+4]': 165, '[Pd]': 50, '[H-]': 72, '[Pt+2]': 145, '[Pt-2]': 241, '[V+3]': 405, '[W+6]': 406, '[13C@H]': 482, '[Pd+4]': 472, '[24NaH]': 572, '[AlH]': 101, '[NH4+]': 65, '[Pd+2]': 82, '[18OH]': 491, '%23': 419,

'[UH]': 554, '[SeH2]': 476, '7': 76, '[LiH]': 213, '[PbH2]': 654, '[15nH]': 523, '[Cl+2]': 271, '[BaH2]': 530, '[Rh+4]': 549, '[Mo+]': 663, '[CH2]': 158, '[La]': 239, '[Li]': 62, '[Al-2]': 671, '[PH3+]': 243, '[99Tc]': 463, '[TaH3]': 517, '[GeH2+]': 614, '[C@]': 55, 'O': 19, '[Cr+2]': 299, '[Rh]': 133, '[AsH2]': 483, '[p-]': 542, '[67Ga]': 585, '[IH3]': 684, '[Ru+3]': 262, '[Si+]': 383, '[Au+]': 400, '[Rh-4]': 626, '3': 26, '[GeH3]': 431, '[AlH-]': 272, 'n': 25, '[N@+]': 362, '[Cr+6]': 290, '[Bi]': 179, '[as]': 657, '[Th]': 296, '[C-]': 86, '[N+2]': 485, '[Re+5]': 543, '[Sc+3]': 214, '[Mg+2]': 106, '[Ho]': 589, '[Sb-]': 219, '[S]': 132, '[te]': 232, '[Hf+4]': 301, '[K+]': 51, '[Ti+]': 360, '[Co-2]': 616, '[SiH]': 97, '[Cu]': 75, '[Ir+]': 375, '[Hg]': 148, '[siH]': 428, '[Cr+4]': 408, '9': 124, '[In-]': 675, '[14C]': 302, '[Sb+]': 515, '[Cl]': 229, '[Hg-]': 668, '[S+2]': 288, '[Sm+2]': 353, '[Mn+]': 553, '[AcH]': 550, '[ClH+]': 409, '[c-]': 66, '[Os]': 125, '[sH+]': 338, '[B-]': 88, '[Fe+2]': 78, '[N]': 177, '[CH+]': 191, '[Ti-2]': 632, '[OH2+]': 197, '4': 32, '[Sc]': 334, '[FeH]': 680, '[RuH]': 417, '/': 39, '[As]': 184, '[unused8]': 8, '[Ce]': 166, '[Na+]': 46, '[Sr]': 351, '[I+2]': 221, '[Sb+5]': 373, '[N+3]': 376, '[Ti+4]': 129, '[Ce+4]': 160, '[BH-]': 81, '[Br+]': 350, '[14CH]': 381, '[Ti+6]': 411, '[BiH3]': 526, '[PH2-]': 688, '[P-]': 67, '[As-]': 322, '[14CH3]': 407, '[U]': 440, '[Bi+2]': 460, '[Ru-2]': 492, '[Yb+2]': 507, '1': 20, '[Ca+]': 547, '[FH+2]': 525, '[XeH]': 652, '[IH2+3]': 681, '[C]': 94, '[In+3]': 266, '[CH3]': 293, '[IrH2]': 512, '[Pt-3]': 603, '[AlH2+]': 524, '[ClH2+]': 445, '[V+4]': 456, '[Ru+]': 254, '[CLS]': 12, '[MgH]': 582, '[Au-3]': 635, '[Fe-3]': 171, '[s+]': 183, '[Au-]': 397, '[Ta-]': 679, '[P@@]': 203, '>>': 29, '[118Sn]': 560, '[Mn+4]': 205, '[CH3-]': 233, '[Cl+]': 138, '[124I]': 425, '[P@@H]': 498, '[Pd+]': 141, '[LaH]': 426, '%24': 450, '[Pr+3]': 374, '[Pm]': 672, '[F+]': 441, '[TeH2]': 478, '[18F]': 172, '[Pb+3]': 278, '[P+2]': 562, '[Ga]': 281, '[unused6]': 6, '[2-benzhydryloxyethyl]': 599, '[Ir+3]': 332, '[unused7]': 7, '=': 22, '[O-]': 36, '[Ga+3]': 345, '[B]': 167, '[Os+2]': 453, '[Fe-2]': 661, '[AlH3]': 333, '%22': 399, '[NiH]': 544, '[S@@]': 111, '[OH]': 263, '[Re+4]': 559, '[Bi+3]': 237, '[F-]': 89, '[Fe-4]': 280, '[Pd+3]': 462, '[Fr]': 573, '[NH3+]': 107, '[Po]': 648, '[Tl-]': 607.

E Robustness verification

To better estimate performance, we report MoleSG's results for 10 seeds in Table 4 and Table 5. From the results, we can observe that the results of ten repeated experiments are close to the results of three repeated experiments, indicating that our model has better robustness.

Table 4. Comparison of the effect of MoleSG between three seeds and ten seeds on classification benchmarks. (Higher values indicate better performance.)

Times \ Dataset	BBBP	Tox21	ToxCast	SIDER	Clintox	BACE	MUV	HIV
3	**97.9±0.3**	**85.0±1.2**	**74.2±0.5**	**70.0±0.2**	**99.1±0.9**	95.1±2.1	**85.1±0.8**	**87.7±1.9**
10	97.9±0.5	84.7±0.8	73.7±0.5	68.0±0.8	97.6±1.0	**95.3±1.6**	83.9±1.1	85.8±1.8

Table 5. Comparison of the effect of MoleSG between three seeds and ten seeds on regression benchmarks. (Lower values indicate better performance.)

Times \ Dataset	ESOL	Freesolv	Lipophilicity	QM7	QM8	QM9
3	**0.599±0.067**	**0.932±0.131**	0.545±0.014	**29.6±2.9**	**0.0118±0.001**	**0.00313±0.00006**
10	0.625±0.038	1.032±0.169	**0.544±0.010**	37.3±5.9	0.0124±0.001	0.00321±0.00007

F The selection of encoder on fine-tuning stage

We have further added new experiments to combine graph and SMILES encoders. We reserve the graph encoder, SMILES encoder and backbone during downstream tasks, and add a prediction head behind the backbone. Since the backbone is pre-trained for mask reconstruction that has a large gap between the downstream tasks, we only try to freeze the graph/SMILES encoder instead of this backbone during downstream tasks. We add a new experiment using the following four different settings. The results in Table 6 and Table 7 show that only using the graph encoder is better than combining the two encoders.

Table 6. Ablation experiments of fine-tuning encoder selection on classification benchmarks. The performance of training all models from scratch is shown in the "Scratch" row. The performances of fine-tuning all pre-trained models, only freezing SMILES encoder, and only freezing graph encoder are shown in "Fine-tune_all", "SMILES_freeze", and "Graph_freeze" rows, respectively. (Higher values indicate better performance.)

Method \ Dataset	BBBP	Tox21	ToxCast	SIDER	Clintox	BACE	MUV	HIV
Scratch	96.4±1.1	83.8±1.8	71.1±1.4	59.1±0.9	84.2±2.4	86.3±1.8	73.0±4.9	78.4±2.7
Fine-tune_all	97.4±0.6	83.0±3.0	71.2±1.0	60.6±1.0	92.4±1.9	87.5±1.2	75.5±2.3	80.3±1.5
SMILES_freeze	97.4±0.8	83.3±0.6	71.4±0.5	62.0±6.9	66.2±30.5	88.0±2.4	75.4±1.5	82.3±3.0
Graph_freeze	96.7±1.6	82.3±1.8	69.9±1.7	61.2±3.1	79.9±3.8	82.4±3.3	68.1±1.2	79.1±0.8
Ours	**97.9±0.3**	**85.0±1.2**	**74.2±0.5**	**70.0±0.2**	**99.1±0.9**	**95.1±2.1**	**85.1±0.8**	**87.7±1.9**

Table 7. Ablation experiments of fine-tuning encoder selection on regression benchmarks. The performance of training all models from scratch is shown in the "Scratch" row. The performances of fine-tuning all pre-trained models, only freezing SMILES encoder, and only freezing graph encoder are shown in "Fine-tune_all", "SMILES_freeze", and "Graph_freeze" rows, respectively. (Lower values indicate better performance.)

Methods \ Dataset	ESOL	Freesolv	Lipophilicity	QM7	QM8	QM9
Scratch	0.812±0.093	1.560±0.217	0.708±0.054	171.3±9.8	0.0130±0.002	0.00723±0.00015
Fine-tune_all	0.782±0.078	1.468±0.229	0.672±0.071	164.2±2.0	0.0124±0.001	0.00653±0.00072
SMILES_freeze	0.822±0.123	1.250±0.491	0.664±0.079	165.3±3.9	0.0131±0.002	0.00613±0.00123
Graph_freeze	0.869±0.036	1.277±0.199	0.878±0.097	166.7±4.0	0.0141±0.001	0.00950±0.00020
Ours	**0.599±0.067**	**0.932±0.131**	**0.545±0.014**	**29.6±2.9**	**0.0118±0.001**	**0.00313±0.00006**

G Learning efficiency analysis

As shown in Figure 1 and Figure 2 below, multi-modality pre-training can slightly speed up the convergence of downstream tasks than single-modality pre-training, showing the advantage over single-modality method in terms of the learning efficiency.

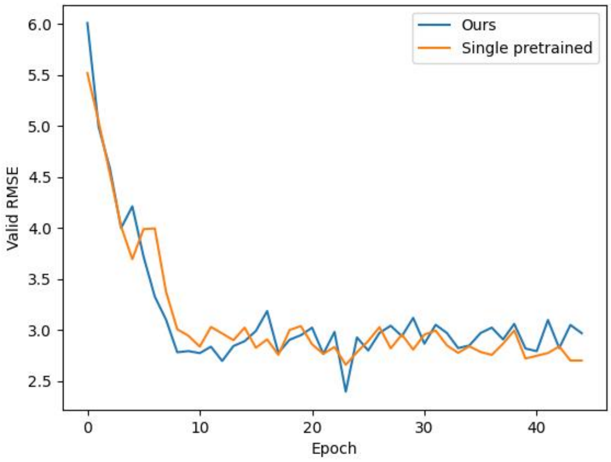

Figure 1. Validation RMSE comparison between models using graph encoders from multi-modality pre-training (Ours) and single-modality pre-training (Single pretrained) on the Freesolv downstream task.

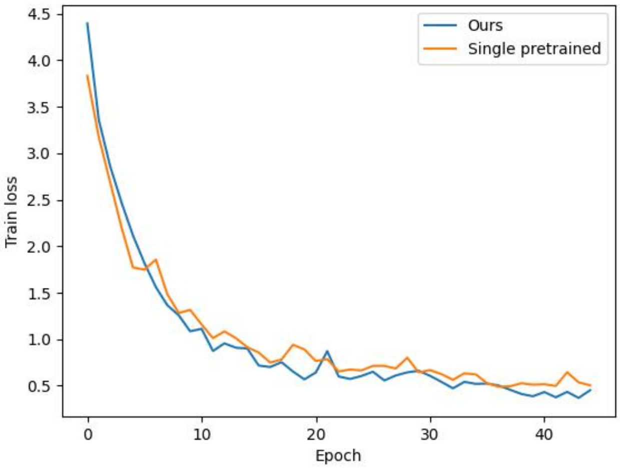

Figure 2. Training loss comparison between models using graph encoders from multi-modality pre-training (Ours) and single-modality pre-training (Single pretrained) on the Freesolv downstream task.

References

[1] Ines Filipa Martins, Ana L Teixeira, Luis Pinheiro, et al. A bayesian approach to in silico blood-brain barrier penetration modeling. Journal of chemical information and modeling, 52(6):1686–1697, 2012.

[2] Thomas Hartung. Toxicology for the twenty-first century. Nature, 460(7252):208–212, 2009.

[3] Ann M Richard, Richard S Judson, Keith A Houck, et al. Toxcast chemical landscape: paving the road to 21st century toxicology. Chemical research in toxicology, 29(8):1225–1251, 2016.

[4] Michael Kuhn, Ivica Letunic, Lars Juhl Jensen,et al. The sider database of drugs and side effects. Nucleic acids research, 44(D1):D1075–D1079, 2016.

[5] Kaitlyn M Gayvert, Neel S Madhukar, and Olivier Elemento. A data-driven approach to predicting successes and failures of clinical trials. Cell chemical biology, 23(10):1294–1301, 2016.

[6] Govindan Subramanian, Bharath Ramsundar, Vijay Pande, et al. Computational modeling of β-secretase 1 (bace-1) inhibitors using ligand based approaches. Journal of chemical information and modeling, 56(10):1936–1949, 2016.

[7] Sebastian G Rohrer and Knut Baumann. Maximum unbiased validation (muv) data sets for virtual screening based on pubchem bioactivity data. Journal of chemical information and modeling, 49(2):169–184, 2009.

[8] Kaspar Riesen and Horst Bunke. Iam graph database repository for graph based pattern recognition and machine learning. In Structural, Syntactic, and Statistical Pattern Recognition: Joint IAPR International Workshop, SSPR & SPR 2008, Orlando, USA, December 4-6, 2008. Proceedings, pages 287–297. Springer, 2008.

[9] John S Delaney. Esol: estimating aqueous solubility directly from molecular structure. Journal of chemical information and computer sciences, 44(3):1000–1005, 2004.

[10] David L Mobley and J Peter Guthrie. Freesolv: a database of experimental and calculated hydration free energies, with input files. Journal of computer-aided molecular design, 28:711–720, 2014.

[11] Anna Gaulton, Louisa J Bellis, A Patricia Bento, et al. Chembl: a large-scale bioactivity database for drug discovery. Nucleic acids research, 40(D1):D1100–D1107, 2012.

[12] Lorenz C Blum and Jean-Louis Reymond. 970 million druglike small molecules for virtual screening in the chemical universe database gdb-13. Journal of the American Chemical Society, 131(25):8732–8733, 2009.

[13] Raghunathan Ramakrishnan, Mia Hartmann, Enrico Tapavicza, et al. Electronic spectra from tddft and machine learning in chemical space. The Journal of chemical physics, 143(8), 2015.

[14] Lars Ruddigkeit, Ruud Van Deursen, Lorenz C Blum, et al. Enumeration of 166 billion organic small molecules in the chemical universe database gdb-17. Journal of chemical information and modeling, 52(11):2864–2875, 2012.

[15] Gilmer J, Schoenholz S S, Riley P F, et al. Neural message passing for quantum chemistry[C]. International conference on machine learning, 2017: 1263-1272.

[16] Yang K, Swanson K, Jin W, et al. Are learned molecular representations ready for prime time?[J], 2019.

[17] Song Y, Zheng S, Niu Z, et al. Communicative Representation Learning on Attributed

Molecular Graphs[C]. IJCAI, 2020: 2831-2838.
[18] Chen J, Zheng S, Song Y, et al. Learning attributed graph representations with communicative message passing transformer[J]. arXiv preprint arXiv:2107.08773, 2021.
[19] Liu S, Demirel M F, Liang Y. N-gram graph: Simple unsupervised representation for graphs, with applications to molecules[J]. Advances in neural information processing systems, 2019, 32.
[20] Hu W, Liu B, Gomes J, et al. Strategies for pre-training graph neural networks[J]. arXiv preprint arXiv:1905.12265, 2019.
[21] Rong Y, Bian Y, Xu T, et al. Self-supervised graph transformer on large-scale molecular data[J]. Advances in Neural Information Processing Systems, 2020, 33: 12559-12571.
[22] Zhang Z, Liu Q, Wang H, et al. Motif-based graph self-supervised learning for molecular property prediction[J]. Advances in Neural Information Processing Systems, 2021, 34: 15870-15882.
[23] Fang X, Liu L, Lei J, et al. Geometry-enhanced molecular representation learning for property prediction[J]. Nature Machine Intelligence, 2022, 4(2): 127-134.
[24] Liu S, Wang H, Liu W, et al. Pre-training molecular graph representation with 3d geometry[J]. arXiv preprint arXiv:2110.07728, 2021.
[25] Wang Y, Wang J, Cao Z, et al. Molecular contrastive learning of representations via graph neural networks[J]. Nature Machine Intelligence, 2022, 4(3): 279-287.
[26] Zhu J, Xia Y, Wu L, et al. Dual-view Molecular Pre-training[C]. Proceedings of the 29th ACM SIGKDD Conference on Knowledge Discovery and Data Mining, 2023: 3615-3627.
[27] Xia J, Zhao C, Hu B, et al. Mole-bert: Rethinking pre-training graph neural networks for molecules[C]//The Eleventh International Conference on Learning Representations. 2022.
[28] Fang Y, Zhang Q, Zhang N, et al. Knowledge graph-enhanced molecular contrastive learning with functional prompt[J]. Nature Machine Intelligence, 2023: 1-12.
[29] Guo Z, Yu W, Zhang C, et al. GraSeq: graph and sequence fusion learning for molecular property prediction[C]//Proceedings of the 29th ACM international conference on information & knowledge management. 2020: 435-443.
[30] Pinheiro G A, Da Silva J L F, Quiles M G. Smiclr: Contrastive learning on multiple molecular representations for semisupervised and unsupervised representation learning[J]. Journal of Chemical Information and Modeling, 2022, 62(17): 3948-3960.
